# Emergence of novel domains in proteins

**DOI:** 10.1186/1471-2148-13-47

**Published:** 2013-02-20

**Authors:** Macarena Toll-Riera, M Mar Albà

**Affiliations:** 1 Evolutionary Genomics Group, Research Programme on Biomedical Informatics (GRIB) - Hospital del Mar Research Institute (IMIM), Universitat Pompeu Fabra (UPF), Barcelona, Spain; 2 Catalan Institution for Research and Advanced Studies (ICREA), Barcelona, Spain; 3 Current address: Department of Zoology, University of Oxford, Oxford, UK

**Keywords:** Protein domain, Lineage-specific domain, Evolutionary rate, Novel domain, Gene age, Domain age

## Abstract

**Background:**

Proteins are composed of a combination of discrete, well-defined, sequence domains, associated with specific functions that have arisen at different times during evolutionary history. The emergence of novel domains is related to protein functional diversification and adaptation. But currently little is known about how novel domains arise and how they subsequently evolve.

**Results:**

To gain insights into the impact of recently emerged domains in protein evolution we have identified all human young protein domains that have emerged in approximately the past 550 million years. We have classified them into vertebrate-specific and mammalian-specific groups, and compared them to older domains. We have found 426 different annotated young domains, totalling 995 domain occurrences, which represent about 12.3% of all human domains. We have observed that 61.3% of them arose in newly formed genes, while the remaining 38.7% are found combined with older domains, and have very likely emerged in the context of a previously existing protein. Young domains are preferentially located at the N-terminus of the protein, indicating that, at least in vertebrates, novel functional sequences often emerge there. Furthermore, young domains show significantly higher non-synonymous to synonymous substitution rates than older domains using human and mouse orthologous sequence comparisons. This is also true when we compare young and old domains located in the same protein, suggesting that recently arisen domains tend to evolve in a less constrained manner than older domains.

**Conclusions:**

We conclude that proteins tend to gain domains over time, becoming progressively longer. We show that many proteins are made of domains of different age, and that the fastest evolving parts correspond to the domains that have been acquired more recently.

## Background

Proteins are organized in discrete functional modules called domains [1-3]. Domains are considered independent evolutionary units that have specific functions, fold independently and can combine with other domains in different modular arrangements [3-5]. They have an average length of approximately 120 amino acids [6] and, while short proteins typically contain only one domain, long proteins are usually composed of several domains [1]. Even though most domains have an ancient origin [7], few domain combinations are shared between the three domains of life [3,8]. This indicates extensive reuse of domains during evolution. Proteins can acquire additional domains over time through several mechanisms, including gene fusion, exon extension, exon recombination, intron recombination and retrotransposition [4,9]. Of these, gene fusion has been proposed to be the most important mechanisms in metazoan proteins [10].

Domains that originated in particular lineages are of special interest in helping understand the molecular basis of lineage-specific adaptations [2,5,7,11]. The age of domains can be dated with more precision than that of individual sequences. We can take advantage of the specific patterns of amino acid conservation displayed by each domain type and use sequence profiles or hidden markov models (HMMs) to identify homologues in distant species [12]. Using domain-specific HMMs, Pal and Guda estimated about 40% of human domains had originated in the metazoan or a more recent phylogenetic node [7]. They also found that about 3% of domains corresponded to primate- or human-specific sequences, illustrating the fact that novel domains are continuously formed. The phylogenetic distribution of Pfam domains has also been recently used to date the age of complete proteins from a given species in ProteinHistorian, a dedicated server for the analysis of protein origin [13].

One well-characterized example of lineage-specific domain is the Kruppel-associated box (KRAB), found in several vertebrate species but greatly expanded in mammals. This domain combines with the Zn-finger motif, which is an older domain, and confers strong transcriptional repressor activity to the protein [14]. There is evidence that recently evolved domains are enriched in low-complexity sequences [15], and tend to be more structurally disordered [11], than older domains. An example of a recently formed low-complexity domain is the cornifin domain in the mammalian-specific small proline rich protein (SPRP) family. The repeats in this domain mediate the formation of a thick layer of cross-linked proteins in keratinocytes and thus play a fundamental role in the formation of the skin [16].

In spite of the fact that recently emerged domains are likely to play key roles in the evolution of new protein functions, we still know very little about them. For example it is not known which fraction of young domains is located in newly evolved genes, or which fraction is formed in the context of older - previously existing - genes. Recently originated genes have special characteristics: they are poorly annotated [17,18], they tend to be shorter than average [19,20] and they evolve particularly rapidly [19,21-23]. However, there has been no study to date that compares the evolutionary properties of domains of different age. To address these questions we use domain genome-wide data from human and mouse, and to a lesser extent from *Drosophila*.

## Results

### Identification of recently evolved domains

We obtained from Ensembl a set of 14,599 human genes with 1:1 orthologs in mouse [24]. We used a Pfam collection of domain-specific hidden markov models (HHMs) [25] to identify the domains present in those proteins. We identified 3,465 different protein domains (domain types), corresponding to a total of 21,730 domain occurrences. We classified the domains in three phylogenetic age groups according to their distribution in 15 eukaryotic species (see Materials and Methods): ‘Mammalian’ (mammalian-specific), ‘Vertebrate’ (vertebrate-specific) and ‘Old’ (older). The average number of domain occurrences per domain type increased in older domains, probably reflecting the accumulation of gene duplicates over time. The length of the domains was very similar for domains of different age (Table 1, Additional file 1: Figure S1).

**Table 1:** Domains in mammalian proteins classified by age

**Domain age**	**N domains**	**Domain length**
Old	20,735 (3,039)	145.8 (101)
Vertebrate	916 (363)	157.2 (102)
Mammals	79 (63)	162 (111)

N domains: number of domain occurrences, in brackets different domain types (non-redundant domains). Domain length: mean and median (in brackets) values.

About 12.3% of the domain types only had homologues in species separated from human in the approximately past 550 million years (Mammalian or Vertebrate), suggesting that they had originated relatively recently (Table 1). We obtained a similar number of domains classified in different age groups with varying stringencies in the domain-specific HMM searches (Additional file 1: Table S2), indicating that the results are robust to different HMM search conditions.

Considering the number of proteins containing at least one occurrence of a given domain, the most abundant Old domains were 7 transmembrane receptor, protein kinase and Zn-finger C_2_H_2_ (Table 2). Each of these domains was present in about 3-3.5% of the proteins containing Old domains. When we considered the number of domain occurrences Zn-finger C_2_H_2_ was by far the most abundant domain (Additional file 1: Table S3), with a total of 1,329 domain occurrences (6.4% of the total domain occurrences in human proteins with Old domains). The most abundant Vertebrate domains were the Kruppel-associated box (KRAB) and the SCAN domain, present in 9.2% and 4.5% of the proteins containing Vertebrate domains, respectively. Similar to KRAB, the SCAN domain combines with Zn-finger motifs and exerts regulatory functions [26]. In contrast to Zn-fingers, KRAB and SCAN typically exist as single copy domains. It has been proposed that KRAB domains were recruited into Zn-finger containing proteins in the early vertebrates, whereas SCAN domains appeared later on, just before the split of reptiles and birds [27]. The abundance of these domains in human proteins can be explained by the frequent duplication of genes containing them [28,29].

**Table 2:** List of the most abundant domains in each age group

**Domain**	**N**	**PFAM ID**
**Old**		
7 transmembrane receptor (rhodopsin family)	385	PF00001
Protein kinase domain	339	PF00069
Zinc finger, C_2_H_2_ type	280	PF00096
PH domain	181	PF00169
Homeobox domain	175	PF00046
RNA recognition motif	139	PF00076
Zinc finger, C_3_HC_4_ type	122	PF00097
PDZ domain	120	PF00595
SH3 domain	115	PF00018
Immunoglobulin I-set domain	114	PF07679
**Vertebrate**		
KRAB box	74	PF01352
SCAN domain	33	PF02023
S-100/ICaBP type calcium binding domain	17	PF01023
Small cytokines (intecrine/chemokine), interleukin-8 like	17	PF00048
Mammalian taste receptor protein	11	PF05296
Protein of unknown function	11	PF04826
u-Par/Ly-6 domain	11	PF00021
**Mammalian**		
Transcription elongation factor A	4	PF06137
Intracellular adhesion molecule, N-terminal domain	4	PF03921
Cornifin (SPRR) family	3	PF02389

Note that if the domain appeared more than once in the same protein we counted it as one occurrence.

Domains classified as Mammalian were generally found in a single human protein (and its mammalian orthologues). However, some domains were present in paralogues. One example is the Transcription elongation factor A domain (TF_A/BEX domain), present in a family of transcription factor genes located on chromosome X and which include TCEAL7, a putative tumour suppressor gene, which negatively regulates NF-kappaB mediated pathways [30].

### Gain of recently evolved domains

Recently evolved domains may be parts of new proteins or, alternatively, arise in proteins with an older origin. In order to know which proportion of young domains fall into each of these two categories we dated the age of all proteins in the dataset. We defined the age of each protein by the oldest domain it contained or, if it did not have any known domain (21.7% of proteins), by BLASTP sequence similarity searches against the 15 species proteomes. The majority of young proteins contained no annotated domains (64.3% and 85.2% for Vertebrate and Mammalian proteins, respectively), in contrast to older proteins (only 14% did not contain known domains). We found that the number of domains per protein, as well as the complete protein length, increased with the age of the protein (Table 3). This suggests that, as proteins become older, their length tends to increase through the gain of new domains.

**Table 3:** Evolutionary properties of human proteins of different age

	**Age**	**N proteins**	**Domains/prot.**	Protein length*	**dN/dS**^a*^
Proteins with domains					
	Old	11,039	1.91 (1)	616.3 (473)	0.11 (0.08)
	Vertebrate	473	1.15 (1)	394.3 (269)	0.21 (0.18)
	Mammalian	62	1.02 (1)	267.9 (163)	0.35 (0.33)
Proteins without domains					
	Old	1,816	NA	654.6 (501)	0.15 (0.12)
	Vertebrate	851	NA	449.0 (319)	0.21 (0.18)
	Mammalian	358	NA	308.2 (214.5)	0.39 (0.31)

Mean and median (in brackets) are shown. ^a^Non-synonymous to synonymous substitution rate (dN/dS) was calculated for 10,636 Old, 416 Vertebrate and 40 Mammalian proteins with domains, and for 1,740 Old, 784 Vertebrate and 274 Mammalian proteins without domains. *Kolmogorov-Smirnov test p < 10^-5^ in all pairwise comparisons.

Analysis of the domain content of proteins of different age showed that the majority of young domains have formed in the context of a newly evolved gene. This was true for 50 of the 63 different Mammalian domain types (79.4%) and 234 of the 363 Vertebrate domain types (64.4%). In the remaining cases the younger domain could be found combined with one or more older domains (13 Mammalian and 129 Vertebrate domain types), reflecting either domain fusion events or the emergence of novel domains in existing older proteins.

**Figure 1:**
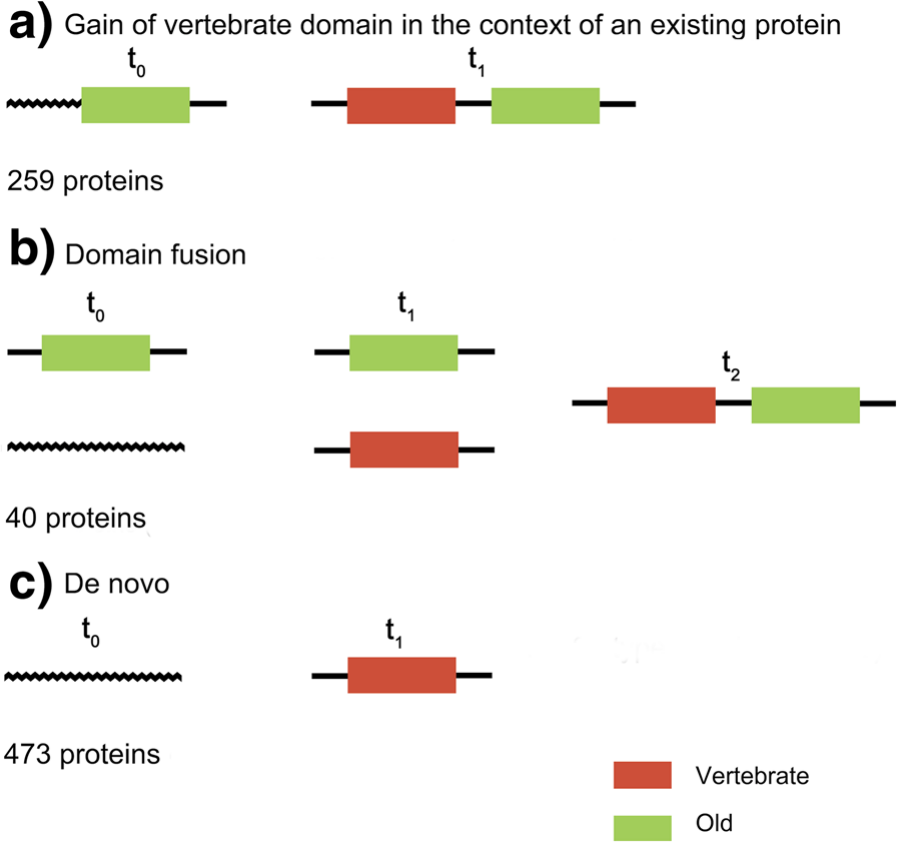
**Evolutionary scenarios for proteins containing Vertebrate domains. a**) The Old domain can be found in combination with the Vertebrate domain as well as separate, but the Vertebrate domain is always found in combination with the Old domain; 118 different Vertebrate domains, 148 different Old domains. **b**) Both Old and Vertebrate domains can be found combined and separately; 11 different Vertebrate domains, 23 different Old domains. **c**) Vertebrate domains that are never found in combination with Old domains; 234 different Vertebrate domains.

In order to investigate the possible importance of domain fusion versus *de novo* domain emergence we examined all possible domain arrangements involving Vertebrate and Old domains present in the 330 human proteins that contained both classes of domain. For each different Old-Vertebrate domain type pair we examined the individual domain occurrences. The largest group was composed of pairs in which the Vertebrate domain only combined with a particular Old domain, whereas the Old domain was found in other domain configurations as well. These cases were compatible with the gain of a novel domain in an existing protein (Figure 1a, 78,5% of proteins). The second most common scenario, albeit much less frequent than the first one, was when we found both the Vertebrate and the Old domain in other domain arrangements, which was compatible with domain fusion (Figure 1b, 12.1% of proteins). For comparison, the number of proteins containing only Vertebrate domains (Figure 1c), probably representing novel vertebrate-specific genes, was about 1.43 times larger than the number of proteins containing both Vertebrate and Old domains (473 *versus* 330 proteins).

**Figure 2:**
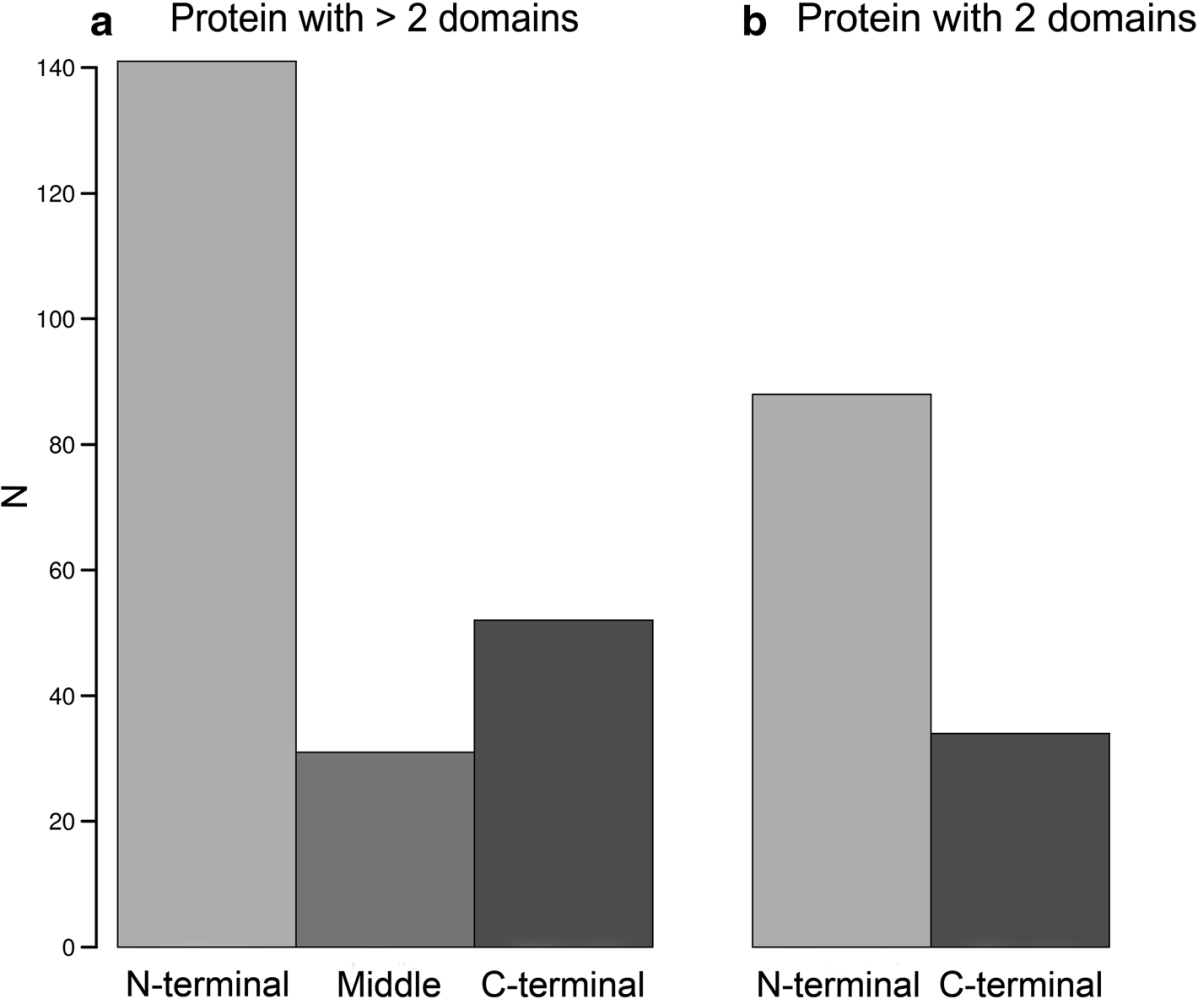
**Relative position of the vertebrate domain in proteins combining old and vertebrate domains. a**. Proteins containing more than 2 domains. **b**. Proteins containing 2 domains.

We next investigated if there was any bias in the localization of Vertebrate domains in proteins also containing older domains. We found a strong bias favouring the incorporation of the Vertebrate domain at the N-terminus both in Old proteins with two domains and in Old proteins with more than two domains (chi-square test, p < 10^-5^, Figure 2). Thus, vertebrate proteins tend to increase in length over time by the gain of recently evolved domains, mainly through the gain or extension of sequences at the 5’end of genes.

### Rapid evolution of young domains independently of protein context

**Figure 3:**
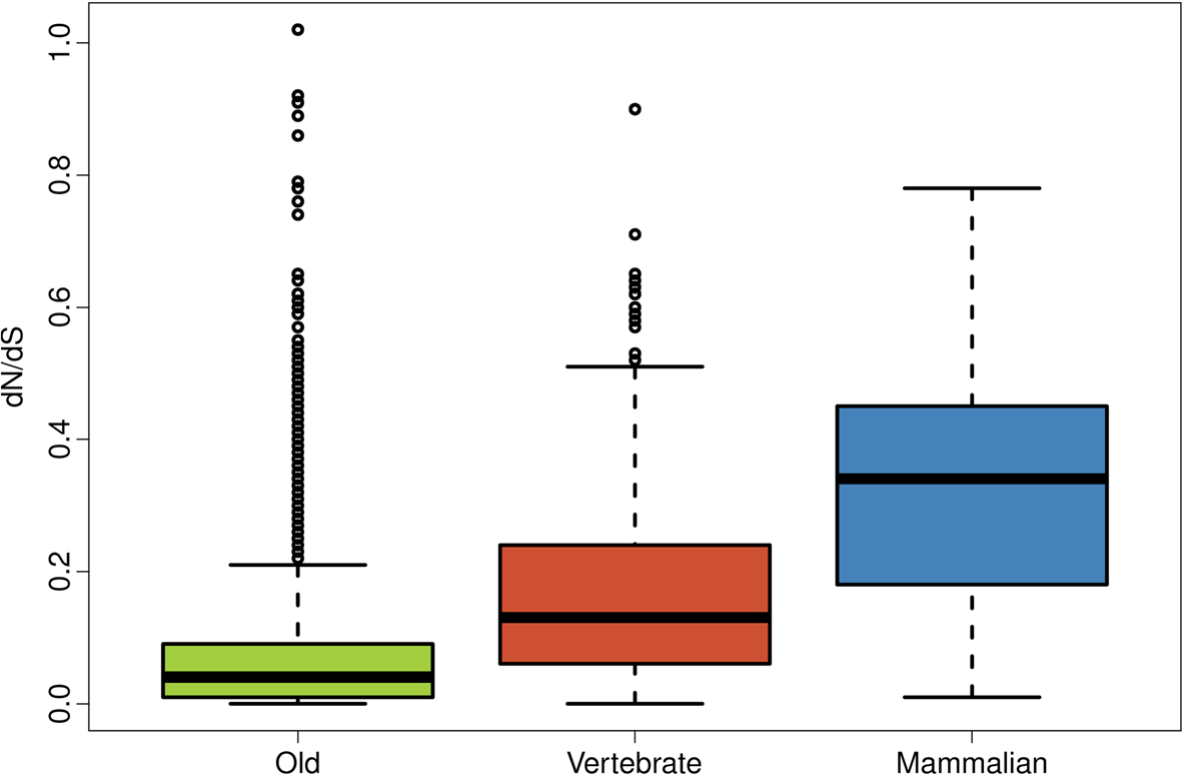
**Non-synonymous to synonymous substitution rate ratio (dN/dS) for protein domains of different age.** Old: 12,076 domains, Vertebrate: 521 domains, Mammalian: 47 domains. dN and dS were calculated for human and mouse orthologous genes, domains with unreliable dN or dS estimates were not considered (domain length < 60 amino acids or dN > 0.5 or dS >2). Differences between each pair of age classes were statistically significant (Kolmogorov-Smirnov test, p-value < 10^-5^). The area within the box contains 50% of the data; horizontal line is the median; outliers (5%) are represented as small circles.

We calculated the non-synonymous to synonymous substitution rate ratio (dN/dS) for all domain regions in human and mouse orthologous proteins. We found that younger domains had significantly higher dN/dS values than older ones (Kolmogorov-Smirnov test, p < 10^-5^) (Figure 3, Additional file 1: Table S4), indicating that they are evolving more rapidly. The results did not vary significantly when we used the median dN/dS for each domain type as the representative domain dN/dS value, which eliminated possible biases caused by very abundant domains (Additional file 1: Figure S5). The results were essentially identical when we employed less stringent E-value cut-offs to classify the domains in different age groups, stressing the robustness of the relationship between domain age and dN/dS (Additional file 1: Table S2).

**Figure 4:**
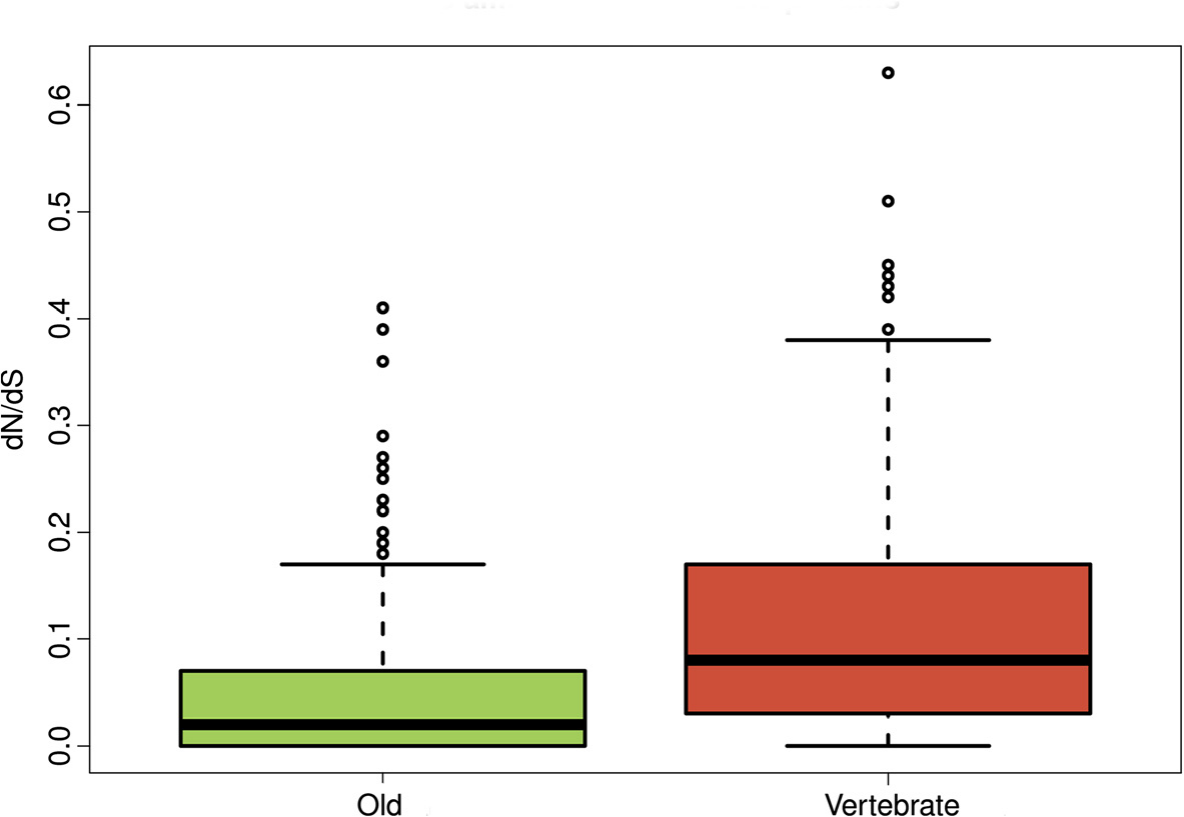
**Distribution of non-synonymous to synonymous (dN/dS) substitution rates for domains of different age combined in the same protein.** dN/dS values were calculated in the domains found in 330 human and mouse 1:1 orthologous proteins. Differences in dN/dS between Old and Vertebrate domains were highly statistically significant (Wilcoxon test, p < 10^-5^).

**Figure 5:**
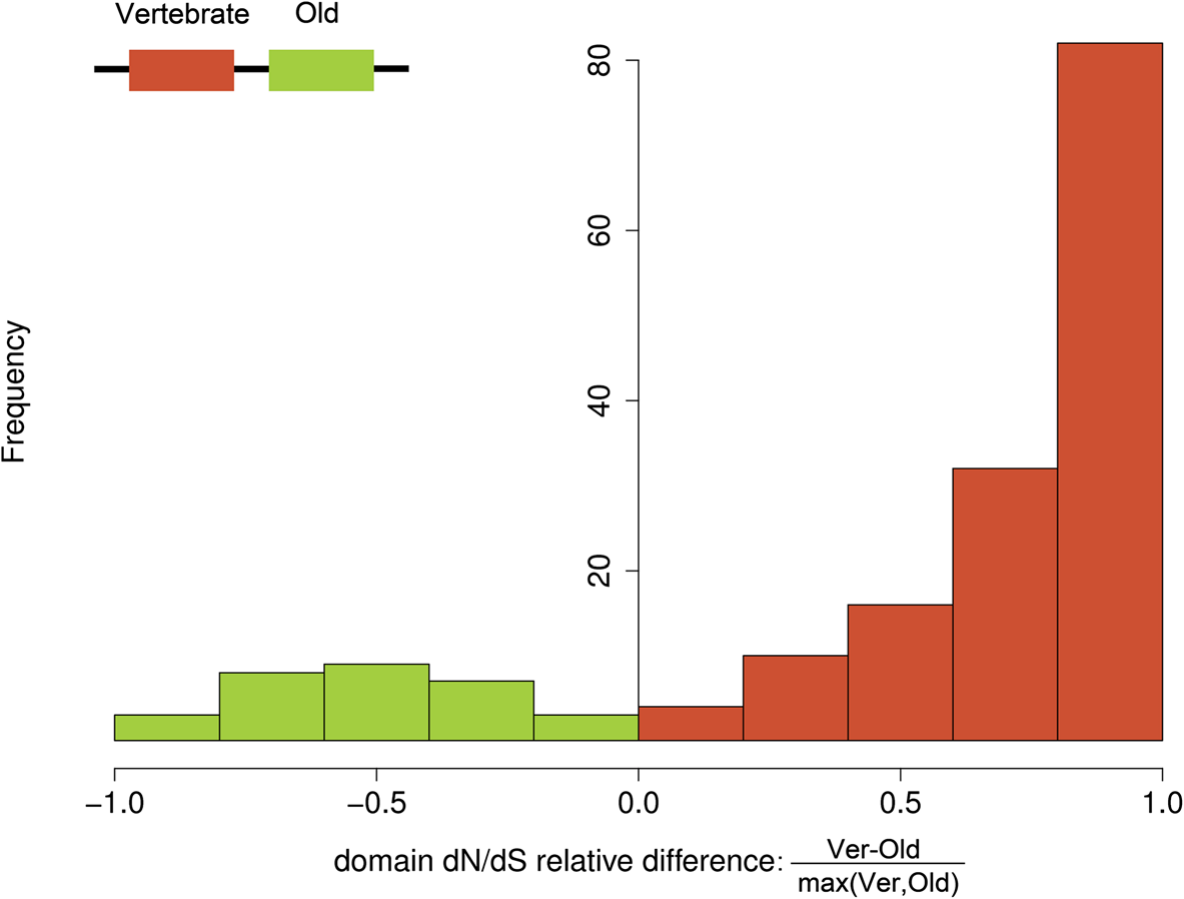
**Differences in dN/dS between vertebrate and old domains located in the same protein.** N = 174 pairs of Old and Vertebrate domains; domain dN/dS relative difference: non-synonymous to synonymous substitution rate ratio (dN/dS) of the Vertebrate domain minus the dN/dS of the Old domain divided by the higher of the two.

In the previous section, we have shown that domains of different age are sometimes found in the same protein. In this situation, do they maintain their characteristic age-related evolutionary rates? To answer this question we focused on the 330 proteins containing both Old and Vertebrate domains. Interestingly, we found that the difference between Old and Vertebrate domains was maintained (Wilcoxon test, p < 10^-5^) (Figure 4). Out of 174 domain pairwise comparisons, 141 showed higher dN/dS values for the Vertebrate domain, compared with only 27 for the Old domain (the remaining 6 cases did not show any significant differences, binomial test, p-value > 0.01). Furthermore, the relative difference in dN/dS values tended to be much larger in pairs in which the Vertebrate domain was evolving faster than when the Old domain was evolving faster (Figure 5).

One example of a protein containing domains of different age is the human progesterone receptor. This protein contains three Pfam domains, an intracellular domain named ‘progesterone receptor’, which is activated by the steroid hormone progesterone, a Zn finger domain, and an extracellular ligand-binding domain. The first domain is vertebrate-specific, and has a dN/dS of 0.2, whereas the other two domains, which are older, have dN/dS values of 0.001 and 0.023, respectively. This illustrates the patterns observed in many proteins, in which the younger parts are evolving much more rapidly. This and other examples are shown in Additional file 1: Figure S6. A complete list of annotated proteins with Old and Vertebrate domains is provided in Additional file 2.

## Discussion

Domains typically cover the majority of a protein sequence and play a crucial role in protein evolution. The way different domains combine, and the mechanisms of domain gain in proteins, have been thoroughly studied [1,3-6,9,11,31]. Other works have described the existence of domains of different age and the relationship of recently evolved domains with lineage-specific innovations [2,7,11]. Here we have focused on the evolutionary properties of young domains to better understand which is their impact on the evolution of the complete proteome. We have found that about two thirds of the young (vertebrate- or mammalian-specific) domains are located in newly evolved genes and the rest arose in already existing proteins. Young domains are preferentially found at the proteins ends, more often at the N-terminus than at the C-terminus, although the reasons for this latter bias are yet unclear. The addition of young domains to already existing proteins is likely to contribute to an increase in protein sequence length over time. We have also discovered that younger domains tend to evolve significantly faster than older domains, even when located in the same protein.

Among young domains, we have been able to identify many less mammalian-specific (Mammalian) domains than vertebrate-specific (Vertebrate) domains (63 *versus* 363). This is not surprising given that the number of Mammalian proteins is about one third the number of Vertebrate proteins. In addition, the percentage of Mammalian proteins with annotated domains is less than half the corresponding percentage of Vertebrate proteins. As the length of the two periods considered is not very different, this very likely reflects strong under-annotation of mammalian-specific sequences in the databases, both in relation to the number of expressed genes and to the number of functional domains in the encoded proteins. In line with this, Capra and colleagues found that younger proteins in yeast were less well covered by Pfam domains than average [17].

Proteins lacking annotated domains show a slight tendency to be longer and evolve more rapidly than proteins containing annotated domains (Table 3). The same characteristics have been previously attributed to proteins with low-complexity regions (LCRs), which undergo continuous repeat expansions and are associated with high mutational dynamics [32]. Therefore, one possible explanation of the data in Table 3 is differences in LCR content [15]. We confirm such differences: the average fraction of the protein covered by LCRs is 11.76 for proteins without domains and 9.34 for proteins with domains (median 8.58 and 6.4, respectively, Kolmogorov-Smirnov test p < 10^-5^). In addition, the underrepresentation of domains in younger proteins is consistent with the previous finding that younger proteins are enriched in LCRs [15]. In conclusion, a large part of the variation in length and evolutionary rate is probably related to differences in LCR content. However, we cannot completely rule out the possibility that some proteins lacking annotated domains have escaped domain detection due to very fast sequence divergence.

We identify 330 proteins in which Vertebrate and Old domains combine. The fraction of Vertebrate domains that belong to Pfam clans (groups of evolutionary related domains) is much smaller for Vertebrate domains than for Old domains (14% vs 59%), emphasizing the recent origin of most Vertebrate domains. In general, novel domains in proteins can be gained by several mechanisms, such as gene fusion, exon extension, recombination and retro-transposition [4,9,33]. It has been hypothesised that domain architecture in all branches of life tends to gain in complexity over time, with a preponderance of fusion events over other types of rearrangements [10,34]. We have observed that Vertebrate domains tend to exist in a single configuration, showing a strong dependence for a given Old domain (or combination of Old domains). In contrast, Old domains from these proteins can also be found in proteins that lack any Vertebrate domain. This provides strong evidence for protein extension as the main mechanism of gain of newly evolved domains in existing proteins (Figure 1). This may be mediated by different mechanisms such as the cooption of adjacent non-coding sequences (exon extension), expansion of repetitive sequences by slippage [35] or insertion of sequences derived from retrotransposons [27]. The gain of new domains in existing proteins, together with the finding that old proteins contain more domains than younger ones (Table 3), is consistent with a scenario in which proteins tend to become more complex over time with regards to the number of different functional domains they contain.

Newly evolved domains are predominantly gained at the N-terminus and, to a lesser extent, at the C-terminus. Diverse authors have found that both domain gain and loss are more frequent at the protein termini than at the protein central region [10,33,36,37]. This may be expected given that the protein ends tend to be more flexible, charged and located at the protein surface than other regions [6]. However, a strong bias towards the N-terminus has not been documented previously, perhaps because it is a special feature of recently evolved domains.

Younger proteins have been found to evolve more rapidly than older proteins in a variety of organisms, including bacteria [38], *Drosophila*[19], mammals [21], yeast [22] and primates [20]. Here we have demonstrated that this age-related effect also applies to protein domains, with younger domains showing higher non-synonymous to synonymous substitution rate ratios (dN/dS) than older ones (Figure 3). In addition, we observe a similar relationship in *Drosophila* domains of different age (Additional file 1: Figure S7 and Table S8), indicating that the observed property is likely to be universal. Therefore younger domains can diverge much more rapidly than older ones, probably mainly due to relaxed selective constraints, as shown to be the case for recently arisen complete coding sequences [23]. It is remarkable that young domains found in otherwise highly conserved proteins (containing Old domains) also evolve very rapidly, further stressing the importance that the time elapsed since a protein sequence originated has on its evolutionary rate.

This work highlights the importance of recently evolved domains in the ongoing evolution of proteins. It shows that proteins should be considered heterogeneous entities in which sequences formed at different times maintain their characteristic evolutionary signatures. The expected future characterization of a larger number of lineage-specific proteins and their functional domains will help shed more light on the early stages of domain evolution.

## Conclusions

The identification of protein domains of recent evolutionary origin is crucial to understand species and lineage-specific adaptations, but these domains are still poorly characterized. In order to fill this gap we have compared the evolutionary properties of human protein domains of different age: mammalian-specific, vertebrate-specific and older. We have found that when domains of different age combine in the same protein the younger domain tends to evolve much faster than the older domain, reinforcing the idea that the time elapsed since a sequence originated largely determines its current evolutionary rate.

## Methods

### Protein domain identification

We obtained 15,630 one-to-one orthologous human and mouse genes using version 56 of Ensembl [24]. We took the protein corresponding to the longest coding transcript for each gene as representative, as defined in Ensembl. We used Hmmpfam (HMMER 2.3.2) [12] to identify all known protein domains in the human and mouse proteins with an E-value cut-off of 10^-5^. We employed the Pfam_ls (version 23) library, which contains 10,340 hidden markov models derived from Pfam domains [25]. We used an in-house Perl program to parse the Hmmpfam results and to assign the domains to the proteins. We identified 3,482 different domains in 14,784 human proteins with 1:1 orthologs in mouse. The results are available from Additional file 3.

### Determination of the age of protein domains

To classify human domains into age groups we used the following classes: mammals (*Mus musculus*, *Rattus norvegicus, Bos Taurus),* non-mammalian vertebrates *(Danio rerio*, *Gallus gallus*, *Takifugu rubripes*, *Xenopus tropicalis*), other metazoans (*Anopheles gambiae*, *Caenorhabditis elegans*, *Ciona intestinalis*, *Drosophila melanogaster)* and other eukaryotes (*Arabidopsis thaliana, Oryza sativa, Saccharomyces cerevisiae*, *Schizosaccharomyces pombe)*. We assigned an age group to each domain following the rank of species in which a domain was found, allowing for secondary losses. For example if a human domain was found in at least one mammalian species but in none of the other vertebrate, metazoan or eukaryotic species it was classified as Mammalian. We classified 2,294 different human domains as Eukarya, 745 as Metazoan, 369 as Vertebrate and 65 as Mammalian. The Eukarya and Metazoan groups were both considered Old (older than 550 million years) and merged into a single class (Tables 1 and 2). Using less stringent E-value cut-offs we obtained similar results in the classification of domains (Additional file 1: Table S2).

### Determination of the age of proteins

We defined the phylogenetic age of a protein as equal to the oldest domain it contained. We obtained 11,039 proteins classified as Old, 473 as Vertebrate and 62 as Mammalian (Table 3). The dataset contained 3,088 proteins that did not have any domain. For these proteins we used BLASTP sequence similarity searches against the genomes listed before to classify them in phylogenetic age groups (E-value < 10^-4^) [39]. Following this procedure we obtained 1,816 proteins classified as Old, 851 as Vertebrate and 358 as Mammalian (Table 3).

### Estimation of evolutionary rates

We aligned orthologous amino acid sequences using T-coffee [40]. To make sure that we were aligning orthologous domains, we focused on orthologues for which the domain structure was completely conserved between human and mouse, which resulted in alignments of 18,193 orthologous domain pairs. Subsequently, we obtained nucleotide coding sequence alignments based on the T-coffee protein alignments using an in-house Perl program.

For each pairwise human and mouse alignment, we estimated the number of non-synonymous substitutions per non-synonymous site (dN), the number of synonymous substitutions per synonymous site (dS), and the dN/dS ratio. We used the maximum likelihood approach implemented in the codeml program of the PAML software package [41].

Domains shorter than 60 amino acids or with a dN > 0.5 or dS > 2 were discarded to ensure robustness in the evolutionary rate estimation. After the filtering process we obtained 12,647 different human domains with dN and dS data. We observed that Eukarya and Metazoan showed a very similar dN/dS distribution and for this reason we considered them as a single group (Old) in all analyses presented here.

### Comparisons of evolutionary rates from pairs of domains located in the same protein

We compared the non-synonymous to synonymous substitution rates (dN/dS) of pairs of Old and Vertebrate domains located in the same protein (330 proteins). We computed the difference in dN/dS of the Vertebrate domain minus the dN/dS of the Old domain and divided it by the higher dN/dS of the two. To determine if the difference in the estimated number of non-synonymous substitutions to synonymous substitutions was statistically different between Old and Vertebrate domains we applied a binomial test comparing the total number of non-synonymous substitutions and synonymous substitutions between the two age groups.

### Distribution of domains located in the same protein

We assigned each pair of domain types Vertebrate-Old in the 330 proteins (see section above) to one of the following classes: 1. Vertebrate domain dependence on a given Old domain: when the Old domain, but not the Vertebrate domain, could be found in a different domain configuration (243 proteins, 115 Vertebrate domain types); 2. Vertebrate and Old domain dependence on each other: when neither the Old domain nor the Vertebrate domain could be found in a different configuration (16 proteins, 13 Vertebrate domain types); 3. Domain fusion: when both the Old and the Vertebrate domain could be found in a different domain configuration (40 proteins, 10 Vertebrate domain types); 4. Complex: when there were more than two domains in a protein and the different Old-Vertebrate pairs showed a different behaviour (31 proteins). Cases in 1 and 2 were considered to be compatible with the gain of a Vertebrate domain into an existing older protein (259 proteins). No cases where found of “Old domain dependence on a given Vertebrate domain”.

### Statistical tests and graphics

The R statistical software package [42] was used to perform all statistical tests and generate graphics.

## Competing interests

The authors declare that they have no competing interests.

## Authors' contributions

MT-R and MMA designed the study. MT-R performed the computational analyses. MT-R and MMA wrote the manuscript. Both authors read and approved the final manuscript.

## Supplementary Material

Additional file 1: Figure S1 Length distribution of domains of different age. **Table S2.** Non-synonymous to synonymous (dN/dS) substitution rates for domains classified in different age classes defined using different Hmmpfam E-value cut-offs. **Table S3.** List of the most abundant domains in each age group by total number of domain occurrences. **Table S4.** Relationship between evolutionary rates and protein domain age. **Figure S5.** Distribution of the non-synonymous to synonymous (dN/dS) substitution rates for each domain type. **Figure S6.** Examples of human proteins containing Vertebrate and Old domains. **Figure S7.** Distribution of non-synonymous to synonymous (dN/dS) values for *D. melanogaster* protein domains classified in different age groups. **Table S8.** Relationship between evolutionary rate and protein domain age in *D. melanogaster* proteins. Click here for file

Additional file 2 Additional Pfam annotations for proteins containing Pfam domains of different age, including Clan Accession number, Clan Identifier and Pfam Description. Click here for file

Additional file 3 Complete list of proteins and Pfam domains employed in this study, including Ensembl Protein identifier, Pfam Accession number, age class, sequence length and non-synonymous (dN) and synonymous (dS) substitution rates. Click here for file
