# Supplementary material for: Emergence of novel domains in proteins
Source: BMC Evol Biol. 2013 Feb 20;13:47. doi: 10.1186/1471-2148-13-47 (PMC3599535; doi:10.1186/1471-2148-13-47)
Supplement: Additional file 1: Figure S1 — Length distribution of domains of different age. Table S2. Non-synonymous to synonymous (dN/dS) substitution rates for domains classified in different age classes defined using different Hmmpfam E-value cut-offs. Table S3. List of the most abundant domains in each age group by total number of domain occurrences. Table S4. Relationship between evolutionary rates and protein domain age. Figure S5. Distribution of the non-synonymous to synonymous (dN/dS) substitution rates for each domain type. Figure S6. Examples of human proteins containing Vertebrate and Old domains. Figure S7. Distribution of non-synonymous to synonymous (dN/dS) values for D. melanogaster protein domains classified in different age groups. Table S8. Relationship between evolutionary rate and protein domain age in D. melanogaster proteins. [file 1471-2148-13-47-S1.pdf]

**SUPPLEMENTARY INFORMATION**

Toll-Riera, M. and Albà, M.M. Impact of recently evolved domains in protein evolution.

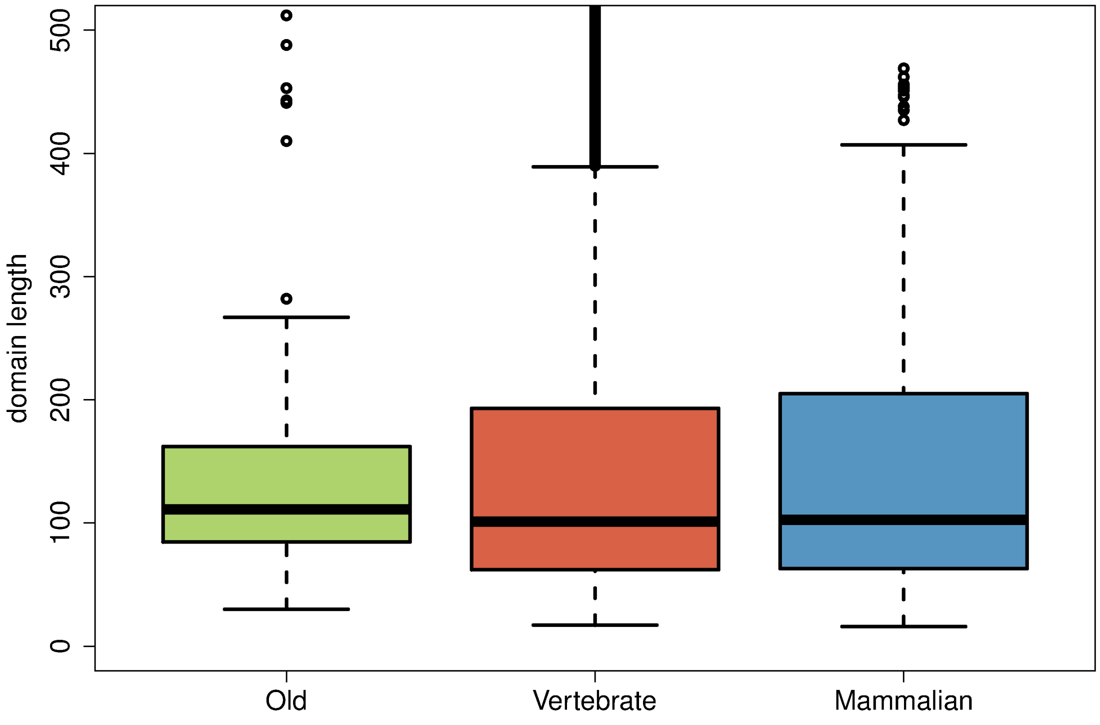


**S1. Length distribution of domains of different age.** The area within the box contains 50% of the data; horizontal line is the median. Outliers (5%) are represented as small circles. The data corresponds to Table 1 in main manuscript file.

| E-value | Age | N | N after filtering | Average dN/dS | Median dN/dS |
|---|---|---|---|---|---|
| 0.00001 | Old | 3,039 | 2,586 | 0.07 | 0.05 |
| | Vert | 369 | 268 | 0.17 | 0.14 |
| | Mam | 65 | 37 | 0.33 | 0.34 |
| 0.001 | Old | 3,069 | 2,605 | 0.07 | 0.05 |
| | Vert | 355 | 256 | 0.18 | 0.14 |
| | Mam | 53 | 29 | 0.32 | 0.34 |
| 0.01 | Old | 3,108 | 2,629 | 0.07 | 0.05 |
| | Vert | 328 | 256 | 0.18 | 0.14 |
| | Mam | 41 | 21 | 0.31 | 0.34 |

**S2. Non-synonymous to synonymous (dN/dS) substitution rates for domains classified in different age classes defined using different Hmmpfam E-value cut-offs.** The default E-value was 0.00001 ($10^{-5}$). N after filtering refers to the number of domain types left after filtering cases with no reliable dN and/or dS measurements (see Materials and Methods in main manuscript text).

| Domain | N | PFAM ID |
|---|---|---|
| **Old** | | |
| Zinc finger, $C_2H_2$ type | 1329 | PF00096 |
| Cadherin domain | 438 | PF00028 |
| Fibronectin type III domain | 387 | PF00041 |
| 7 transmembrane receptor (rhodopsin family) | 385 | PF00001 |
| Protein kinase domain | 348 | PF00069 |
| Immunoglobulin I-set domain | 320 | PF07679 |
| EGF-like domain | 233 | PF00008 |
| RNA recognition motif | 229 | PF00076 |
| Calcium binding EGF domain | 215 | PF07645 |
| Sushi domain (SCR repeat) | 213 | PF00084 |
| **Vertebrate** | | |
| KRAB box | 75 | PF01352 |
| SCAN domain | 33 | PF02023 |
| PKD domains | 19 | PF00801 |
| S-100/ICaBP type calcium binding domain | 17 | PF01023 |
| Small cytokines (intecrine/chemokine), interleukin-8 like | 17 | PF00048 |
| **Mammalian** | | |
| Transcription elongation factor A | 4 | PF06137 |
| Intracellular adhesion molecule, N-terminal domain | 4 | PF03921 |
| Cornifin (SPRR) family | 3 | PF02389 |

**S3. List of the most abundant domains in each age group by total number of domain occurrences.**

| Age | N domain occurrences | N domain types | Average | dN/dS | dN | dS |
|---|---|---|---|---|---|---|
| Old | 12,076 | 2,586 | Mean | 0.07 | 0.05 | 0.75 |
| | | | Median | 0.05 | 0.03 | 0.66 |
| Vertebrate | 521 | 268 | Mean | 0.17 | 0.12 | 0.80 |
| | | | Median | 0.14 | 0.10 | 0.74 |
| Mammalian | 47 | 37 | Mean | 0.33 | 0.24 | 0.80 |
| | | | Median | 0.34 | 0.25 | 0.76 |

**S4. Relationship between evolutionary rates and protein domain age**. Non-synonymous (dN) and synonymous (dS) substitution rates, calculated for human and mouse orthologous sequenes, corresponding to domains classified in different age classes (Old, Vertebrate, Mammalian). dN and dS statistics are calculated for all domain ocurrences. N domain types refer to the number of non-redundant domains. The number of domains analyzed is lower than in S2 because we filtered out domains with unreliable dN or dS estimates (domains shorter than 60 amino acids or with dN>0.5 or with dS>2).

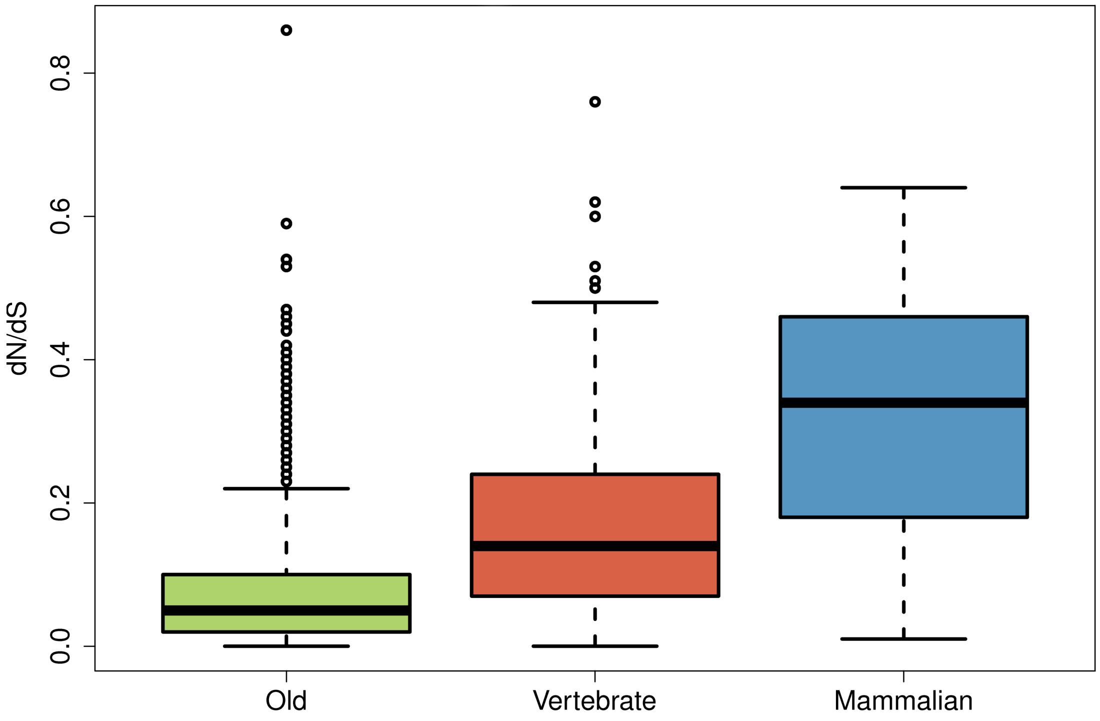


**S5. Distribution of the non-synonymous to synonymous (dN/dS) substitution rates for each domain type.** The median values calculated from the domain occurrences of each domain type were used as data points. The area within the box contains 50% of the data; horizontal line is the median. Outliers (5%) are represented as small circles. Differences between pairs of groups are highly significant (Kolmogorov-Smirnov test, $p<10^{-5}$). The data corresponds to 2,586 Old domains, 268 Vertebrate domains and 37 Mammalian domains.

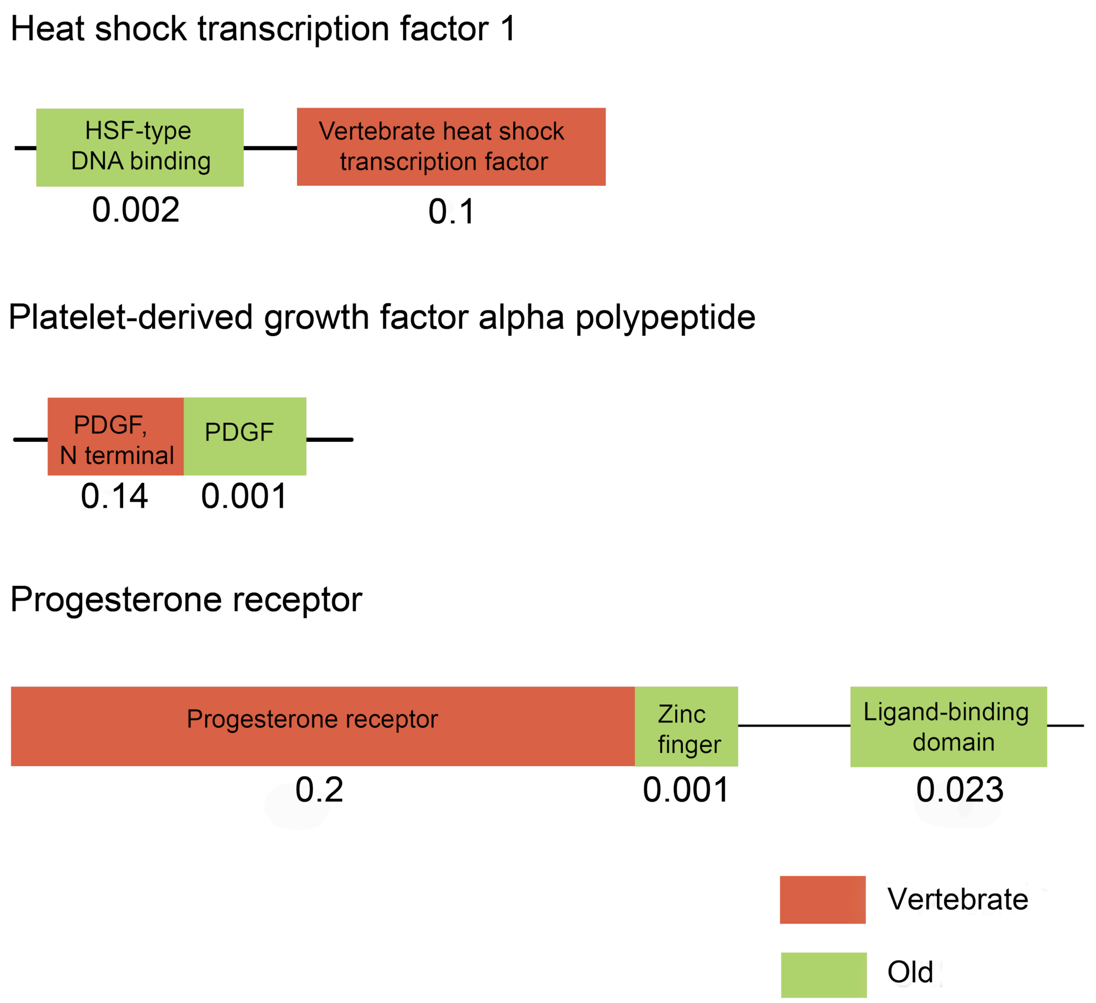


**S6. Examples of human proteins containing Vertebrate and Old domains.** Numbers indicate the non-synonymous to synonymous substitution rate (dN/dS) ratio calculated in human and mouse orthologous sequences. Heat shock transcription factor 1: ENSP00000332698; platelet-derived growth factor alpha polypeptide: ENSP00000346508; progesterone receptor: ENSP00000325120.

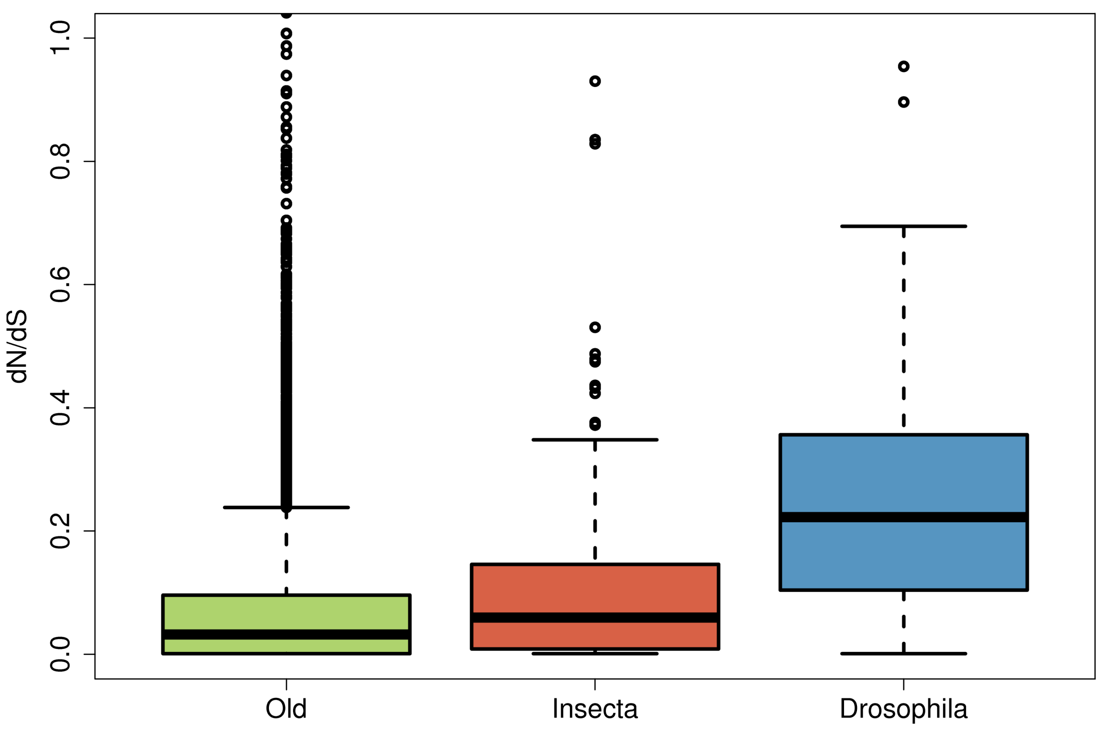


**S7. Distribution of non-synonymous to synonymous (dN/dS) values for *D.melanogaster* protein domains classified in different age groups**. Differences between pairs of age groups are highly significant (Kolmogorov-Smirnov test, $p<10^{-5}$). We obtained 11,013 one to one orthologous genes from *D.melanogaster* and *D.simulans*, using Ensembl (Hubbard et al., 2009). Using domain searches in other proteomes we classified D.melanogaster domains in the following groups: Drosophilids (D. simulans, D. yakuba, D. erecta, D. pseudobscura, D. viriliae, D. Grishewi), non-Drosophila Insecta (Anopheles gambiae, Apis mellifera, Acyrthosiphon pisum), other metazoans (Takifugu rubripes, Homo sapiens, Ciona intestinalis, C.elegans) and other eukaryotes (*Arabidopsis thaliana, Oryza sativa, Saccharomyces cerevisiae*, *Schizosaccharomyces pombe)*. The proteomes were downloaded from Ensembl (Hubbard et al., 2009) and Uniprot (Jain et al., 2009). We classified 1,994 different D.melanogaster domains as Eukarya, 564 as Metazoan, 30 as Insecta and 22 as Drosophila.

| Age | N domain occurrences | N domain types | Average | dN/dS | dN | dS |
|---|---|---|---|---|---|---|
| Old | 6,844 | 2,185 | Mean | 0.138 | 0.012 | 0.151 |
| | | | Median | 0.032 | 0.004 | 0.138 |
| Insecta | 278 | 25 | Mean | 0.114 | 0.016 | 0.177 |
| | | | Median | 0.059 | 0.009 | 0.158 |
| Drosophila | 66 | 16 | Mean | 0.274 | 0.036 | 0.147 |
| | | | Median | 0.222 | 0.027 | 0.132 |

**S8. Relationship between evolutionary rate and protein domain age in D.melanogaster proteins**. Non-synonymous (dN) and synonymous (dS) substitution rates, calculated for *D.melanogaster* and *D.simulans*, corresponding to domains classified in different age classes (Old, Insecta, Drosophila). dN and dS statistics are calculated for all domain ocurrences. N domain types refer to the number of non-redundant domains.
